# Dynamic Modeling and Control Analysis of a PTES System for Grid Support and Multi-Temperature Industrial Heat Delivery

**DOI:** 10.1021/acs.iecr.5c05434

**Published:** 2026-04-01

**Authors:** Alp Albay, Mehmet Mercangöz

**Affiliations:** Department of Chemical Engineering, 4615 Imperial College London, London SW7 2AZ, U.K.

## Abstract

We present a systems-level dynamic analysis and control strategy for a recuperated supercritical CO_2_ Brayton-cycle pumped thermal energy storage system designed to simultaneously deliver grid energy-storage services and multitemperature heat input to industrial sites. A control-oriented dynamic model is developed in MATLAB/Simulink; turbomachinery submodels and finite-volume heat exchanger models capture the thermodynamic transient behavior. Accordingly, control functions are designed and assessed against four objectives: antisurge control for safe turbomachinery operation, cycle power set point tracking for grid services, inventory control to enable operating-point transitions, and coordinated startup/shutdown sequencing. Simulations demonstrate robust transient performance, with power-tracking settling times of 15–45s. Inventory control enables reliable switching between thermodynamic cycle configurations for multitemperature heat pumping, stabilizing pressures within 48 s and temperatures within 73 s. Together, these results provide ramp-rate constraints relevant to operational scheduling and support the feasibility of PTES as a bridge between variable renewable electricity and industrial thermal demand.

## Graphical abstract

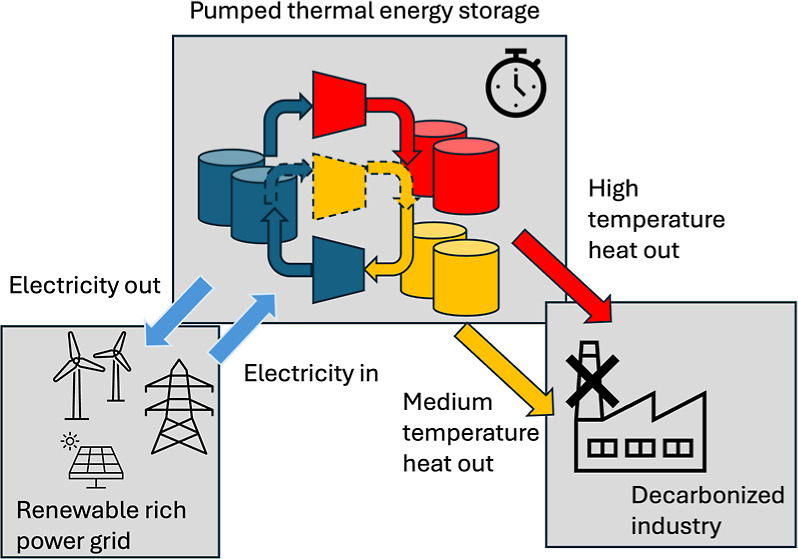

## 1 Introduction

According to the International Energy Agency Net Zero Emissions by 2050 roadmap, the global power generation will be dominated by wind and solar. However, the fast deployment of these variable renewable energy (VRE) sources creates new operational challenges.^1^ Among them two key challenges are pertinent to this study. First key challenge is that power grids that rely heavily on renewables must still cover demand when wind and solar output are low, which is especially problematic during peak demand periods.^2^ This drives the need for scalable and cost-efficient energy storage technologies.^3^ Long duration energy storage (LDES) is a necessary technology for managing variable supply and demand over various time scales (from days to months). Current LDES capacity is composed mostly of pumped hydro storage (PHS).^4^ However, due to geographical constraints, high capital costs, and water scarcity concerns, further development and applications of PHS are limited which requires innovations in scalable energy storage technologies. Second key challenge stems from the fact that while electrification powered by renewable generation is an attractive decarbonization pathway, today’s energy system still depends heavily on conventional thermal technologies for both power-system operability and direct thermal energy supply.^5^ In the power sector, synchronous generators inherently provide inertia and other stabilizing services, which are not naturally available from variable renewable electricity (VRE) resources without additional power-electronic and storage-based solutions.^6^

Outside the power sector, fossil fuels are widely used independently of the grid by being combusted directly at industrial sites to supply process heat across a broad range of temperatures. This highlights a key structural challenge for renewable-led decarbonization: most renewable resources primarily deliver electricity, whereas a large share of end-use demand is thermal. Globally, around 49% of final energy use is thermal,^7^ yet renewables can only reliably supply around 12% of this thermal demand.^8^ The gap is even more pronounced in industry, where approximately 70% of thermal energy is still supplied by fossil fuels,^9^ particularly for medium (100–500 °C) and high (>500 °C) temperature duties. Consequently, there is strong demand for pathways that enable renewable energy to penetrate industrial heat at multiple temperature levels. Figure 1 illustrates selected industrial sectors and the characteristic temperature ranges required by their processes.

**Figure 1:**
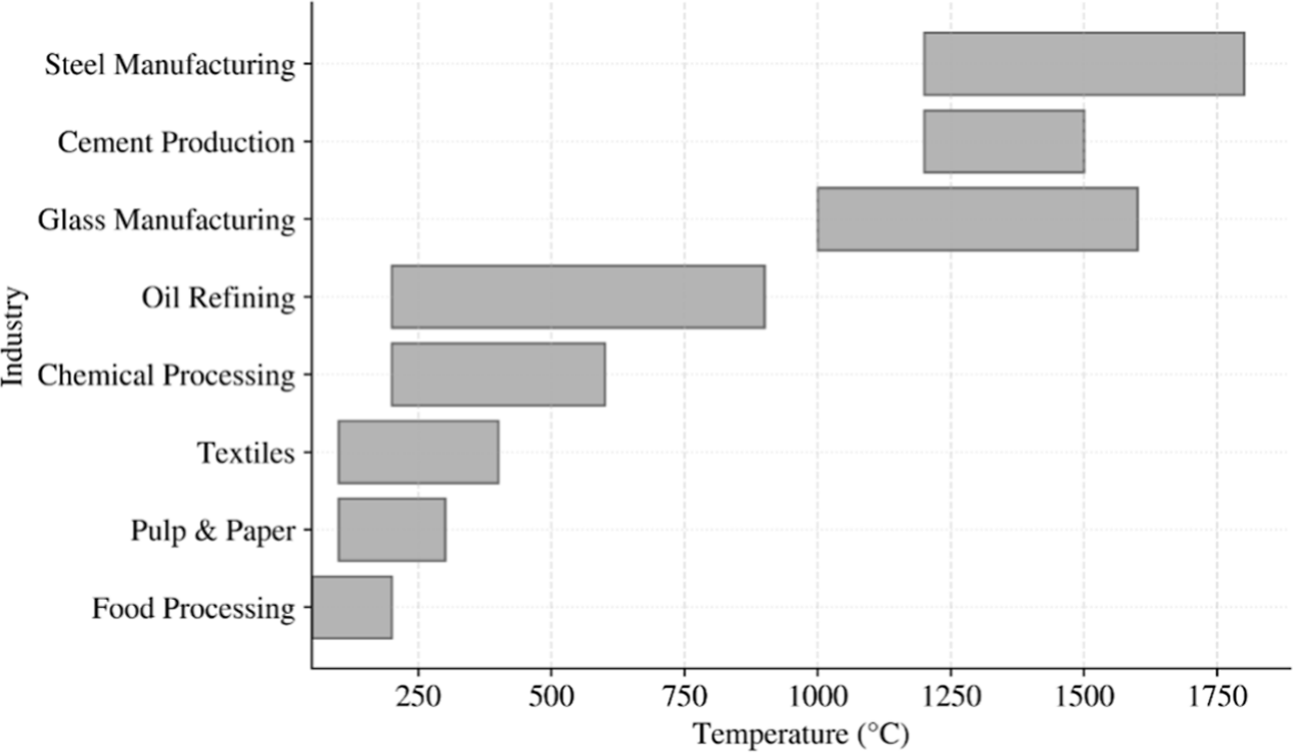
Heating requirements in various industries by temperature (Data for plot collected from^10^).

The aim of this research is to bridge the gap between grid-supporting long-duration energy storage (LDES) and renewable heat demand by framing the problem as a system-level opportunity rather than two separate challenges. In particular, the same industrial and district-heat users that require low-carbon heat can also act as flexible electrical loads: by shifting heat production in time, these sites can absorb surplus renewable electricity and provide demand-side flexibility when the grid is constrained. When a PTES system is coupled with a heat user, the stored energy can be delivered both as heat and electricity. This improves the overall value proposition by offsetting the impact of the low round-trip efficiency of PTES systems. The motivation here is analogous to cogeneration in conventional power generation, where the utilization of primary energy is improved by valorizing discharged heat. Against this backdrop, this article focuses on Pumped Thermal Energy Storage (PTES), which uses a heat pump during charging and a heat engine during discharging with thermal storage media in between. Many underlying thermodynamic cycles have been proposed for PTES;^11^ here, the focus is on a recuperated supercritical CO_2_ Brayton-cycle PTES configuration (rSCBC).

In previous studies, we investigated PTES systems from a variety of angles. In,^12^ a PTES model was developed in ASPEN HYSYS^13^ to study its part-load behavior. Understanding this behavior is essential for integration with variable renewable supplies and grids. This necessitated the development of a state-of-charge management algorithm that maintains equal charge levels in the both the cold and hot storage tanks even when operating at off-design conditions. In addition to this, a scheduling optimization study was carried out, which demonstrated that VRE integrated PTES systems can operate profitably.

Albay et al.,^14^ examined a PTES system designed to supply both heat and electricity while operating alongside VREs and an industrial process. The study showed that although heat can be supplied directly from the hot store, doing so disrupts the required balance between the hot and cold stores. Since the hot-side heat provision bypasses the normal discharge cycle, the cold store must be discharged (i.e., heated) separately. The total round-trip efficiency of the system is increased as the lossy heat engine side is bypassed. This increase is similar to the increase in efficiency in cogenerating power plants.

One drawback of the proposed system in^14^ was that the cold water was discharged using the hot side, meaning valuable high-temperature heat was used to warm a much lower-temperature stream, reducing overall efficiency by lowering the amount of useful energy available for delivery. Also, the coupled process heat requirement temperature and the hot side supply temperature did not match as the study looked at a predesigned PTES system coupled with a process, so the process and system level design was not considered.

This was further built upon in^15^ where a multitemperature storage system was proposed. That study suggested using two additional heat pumps, operating as Joule–Brayton cycles at lower temperatures and pressures, that only store heat and are not used for electricity discharge which draw heat from ambient water. This allows the system to meet a wider range of industrial heat demands without the need for balancing heat in reservoirs. Compared to similarly sized Li-ion batteries coupled with an electric heater, it was 20% more efficient, 40% cheaper and on renewable heavy grids, can provide reliable heat with reduced CO_2_ emissions.

These studies employed a steady-state and a quasi-steady-state formulation of the PTES systems. To understand the behavior fully, any future studies need to investigate the dynamic behavior of such PTES systems to see if they are viable in VRE grid scenarios that require rapid start-up and shut-down times. Dynamic behavior and control of supercritical CO_2_ Brayton cycles employed in power generation cycles are well studied in recent years; Ma et al.^16^ coupled with coal power, Gao et al.^17^ and Conboy et al.^18^ with nuclear power, Ma et al.^19^ with concentrated solar power plants. However, the dynamic control of a PTES system requires the analysis of both the heat pump and heat engine cycles. On this front, Lu et al.^20^ studied the start-up of PTES cycles, while Jiang et al.^21^ worked on the performance and application of printed circuit heat exchangers (PCHE) in PTES systems. An et al.^22^ also look into startup and operating point changes, with a focus on the system temperatures. However, there is no clear study on the transition of PTES systems from one operating cycle to another, which is required for multilevel energy storage.

The objective of this study is to develop and demonstrate a dynamic model and control framework for a PTES system based on a recuperated supercritical CO_2_ Brayton-cycle layout. The work integrates turbomachinery and heat-exchanger dynamic models with a control methodology capable of maintaining stable operation under large transients (start-up/shut-down and thermodynamic cycle changes). Using this framework, we simulate transitions between charging modes that deliver temperature at different levels. This allows us to assess if an existing PTES system can be augmented with these methods to store temperature at multiple levels. This requires testing the models of an existing PTES system, built for specific temperature and pressure ranges, at lower temperatures and pressures. By capturing cycle-to-cycle switching within the dynamic model, the study establishes an operational understanding that can support later techno-economic evaluation and operational scheduling of multitemperature PTES. The main contribution of this work is the development of a plant-wide dynamic modeling and control framework for a multitemperature PTES system, including anti-surge protection, power tracking, inventory-based cycle switching, and startup/shutdown control.

The remainder of this paper is organized as follows. Section 2 describes the PTES system configuration. Section 3 presents the dynamic modeling framework and control design methodology. Section 4 reports the results of the control design study and evaluates the transient performance of the system. Section 5 discusses the implications of these findings for PTES operation and industrial heat integration. Section 6 concludes the paper.

## 2 PTES System Configuration

This section presents the process configuration and operating concept of the PTES system analyzed in this work. The thermodynamic cycle design is first introduced, focusing on the recuperated Brayton-cycle architecture adopted for high-temperature industrial heat integration. The section then discusses the selection of storage materials and temperature levels required to support multitemperature heat delivery. Finally, the operational strategy used to coordinate charging, discharging, and heat-delivery modes is outlined, providing the foundation for the dynamic modeling and control methodology developed in Section 3.

### 2.1 Cycle Design

PTES systems can employ several thermodynamic cycles, which can be broadly categorized as open or closed.^23^ In open-cycle configurations, heat is exchanged directly with the ambient environment, eliminating the need for separate hot and cold storage reservoirs. However, their performance is sensitive to ambient conditions, and the relatively low working-fluid density can lead to large turbomachinery. In addition, the reliance on ambient heat exchange limits the ability to independently control storage temperature levels. Closed-cycle systems, by contrast, require dedicated thermal storage reservoirs but allow the storage temperature levels to be selected according to application requirements and offer greater flexibility in working-fluid choice.

Morandin et al.^24^ and Echogen^25^ have both proposed or developed transcritical Rankine cycles, while McTigue et al.^26^ proposed Joule–Brayton cycles. Transcritical cycles in cited literature typically utilize cryogenic or lower than ambient storages on the cold side and require latent heat storage through phase change materials to match WF evaporation and condensation. By contrast, Brayton-cycle layouts can be designed to remain single-phase throughout, simplifying turbomachinery and operation. Wang and Zhang^27^ analyzed a Rankine cycle layout and found an energy storage density of 22 kWh/m^3^, whereas White et al.^28^ showed Brayton cycles can reach 50 kWh/m^3^. Near-critical Brayton compression can have substantially lower specific compression work than ideal-gas compression because the fluid density is high, which leads to smaller turbomachinery and lower costs.^29^ This benefit arises because WF density increases significantly near the critical point, reducing the specific compression work.^30^ However, the compression temperature/pressure also determines the cold side storage since the compressor inlet/turbine outlet need to be maintained above the critical point.

Since our focus is on industrial heat integration of PTES systems at midto-high delivery temperatures, we consider PTES cycles with high-temperature storage. Therefore, this study adopts a Brayton-based concept with warm heat-source conditions to reduce temperature lift and improve COP compared with low-temperature-source architectures. This section outlines the working principle of a generic recuperated Brayton cycle PTES system. Detailed thermal storage material selection will follow once the fundamentals of the cycle design is established.

Figure 2 illustrates the layout of the PTES system. In the charging cycle (Figure 2a), WF is compressed (6 to 1, Comp) to a high temperature and pressure at which point it then transfers its heat to a hot storage (1 to 2, HHX). Next, the WF exchanges heat with itself in the recuperator, cooling down (2 to 3) before entering the expander (3 to 4, Exp). After the expansion, it is further cooled to a temperature slightly above its critical temperature (4 to 4a). Following the cooler, it enters a cold heat exchanger (CHX), where it is reheated (4a to 5) while cooling the cold storage (9 to 10). It is then preheated in the recuperator (5 to 6) before completing the cycle. The cooler after the expander is there to dump excess heat built up from inefficiencies in the system but also it provides consistency by keeping the cold heat exchanger (CHX) inlet constant. Otherwise, the cold storage temperatures would be variable.

**Figure 2:**
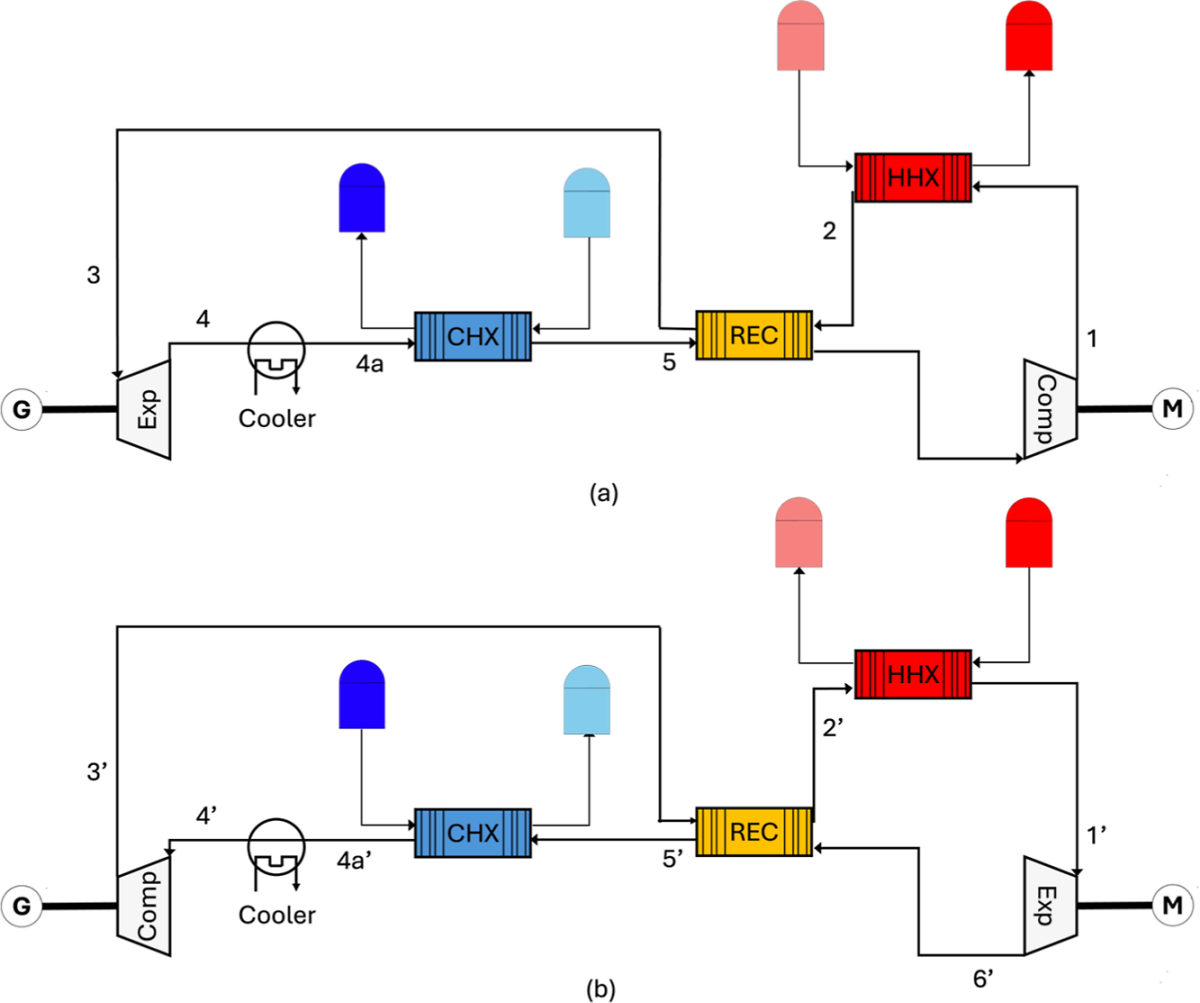
PTES system layout. (a) charging cycle, (b) discharging cycle.

During the discharge process (Figure 2b), the WF receives heat from the hot reservoir through the hot heat exchanger (HHX) (2′ to 1′). It then enters the turbine where it is expanded and generates electricity. After the temperature and pressure drop (1′ to 6′), it is further cooled (6′ to 5′) in the recuperator and enters the CHX, where it dumps heat into the cold storage (5′ to 4a′). It is then cooled even further by the cooler (4a′ to 4′) before entering the compressor, (4′ to 3′). Finally, it completes the cycle by as it is reheated by itself in the recuperator (3′ to 2′).

The recuperator facilitates internal heat transfer and allows for the two storage temperatures to be decoupled from each other. So, each storage temperature becomes a design variable, leading to higher flexibility. The cooler on the low temperature side is included for two reasons: (1) the inefficiencies in the system need to be managed by rejecting heat, (2) the inlet of the CHX and the compressor can be managed with the cooler, which also increases the flexibility of the system.

Heat supply extensions on the original model are based on^14^ and.^15^ Figure 3 shows the two additional heat pumps that are capable of delivering lower temperature heat to different heat storage materials. The three heat pumps can be distinguished as high-, medium- and low-temperature heat pumps. High-temperature heat pump (HHP) is the main heat pump for PTES charging and is coupled with the discharging heat engine cycle. This means that it can be used to discharge both heat and electricity. However, one drawback highlighted in^14^ is that when the hot side is discharged to provide process heat, the cold side remains charged (stays at a low temperature). This necessitates a balance restoring mechanism, which is also shown in Figure 3. The hot storage is used to restore the cold storage without doing work, which results in losses in useable heat.

**Figure 3:**
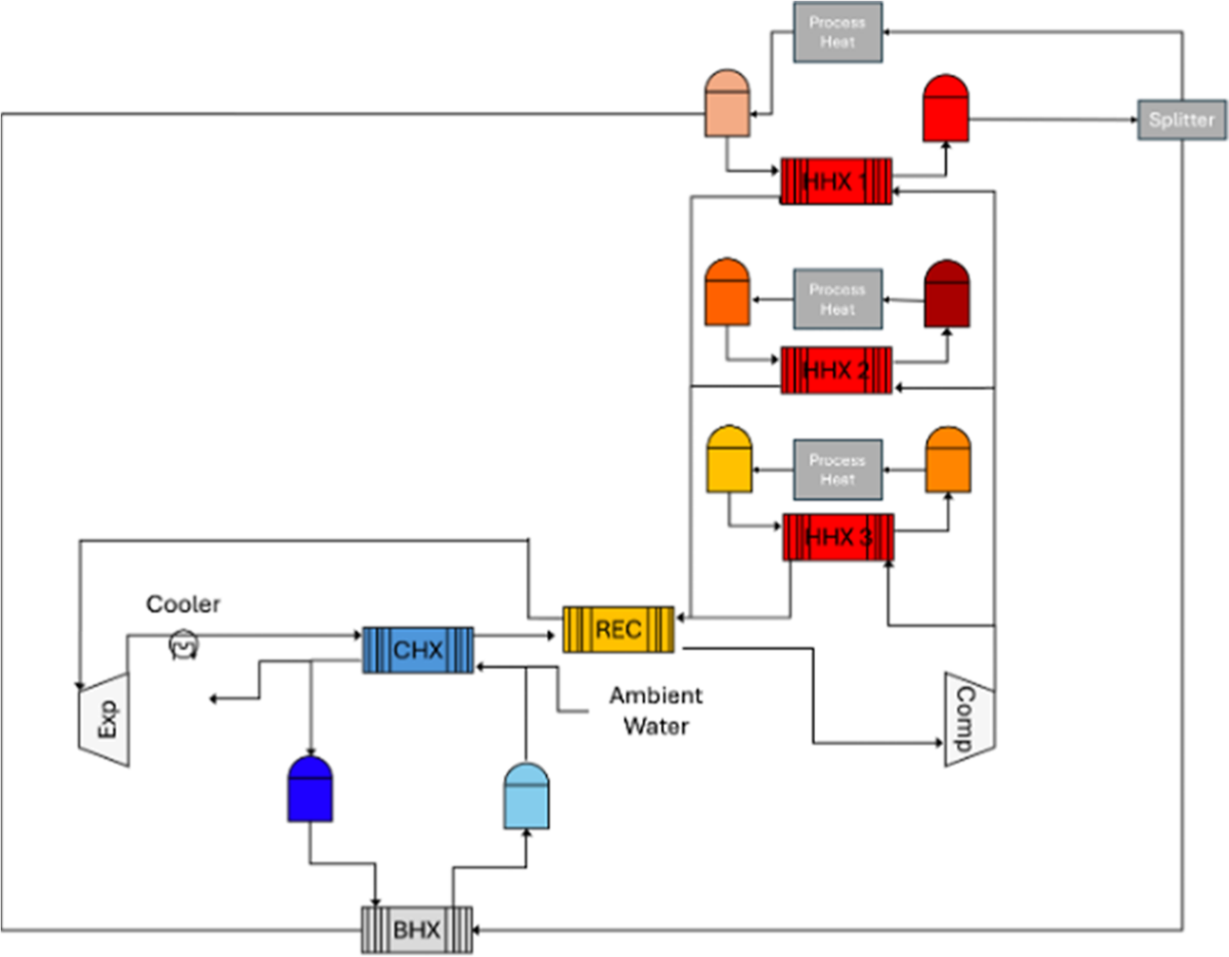
Layout of the additional heat storage equipment for multitemperature energy storage.^15^ HHX 1 is the main heat exchanger for HHP, while HHX 2 and HHX 3 are the heat exchangers for MHP and LHP respectively. BHX is the balance heat exchanger used to discharge the cold side when using heat from the HHP hot storage.

The medium-temperature (MHP) and low-temperature (LHP) heat pumps were designed with this factor in mind. Since they deliver lower temperatures, their heat supply temperature can also be low. This allows for ambient cooling with water or air in an open cycle configuration, while maintaining reasonable COP values. Figure 4 illustrates the different cycles that can be achieved with this layout.^15^ In,^15^ we studied the viability of using the same compressor for all three cycles. They found that if the volumetric flow rate through the compressor remains the same, the behavior of the compressor can still be approximated with the same performance maps. For this reason, when designing the MHP operating conditions, as the overall cycle temperatures drop, the pressure also drops. Since LHP and MHP share the same low temperature side, LHP needs a lower mass flow rate in order to go to lower temperatures in the same pressure region while still keeping the volumetric flow rate constant. This results in three different power ratings for the three heat pumps. This is achieved through precise control of the inventory of the system WF (see Section 4.3) where the mass flow through the system is regulated. This allows for the use of the same compressor at different inlet conditions and heat pump cycles.

**Figure 4:**
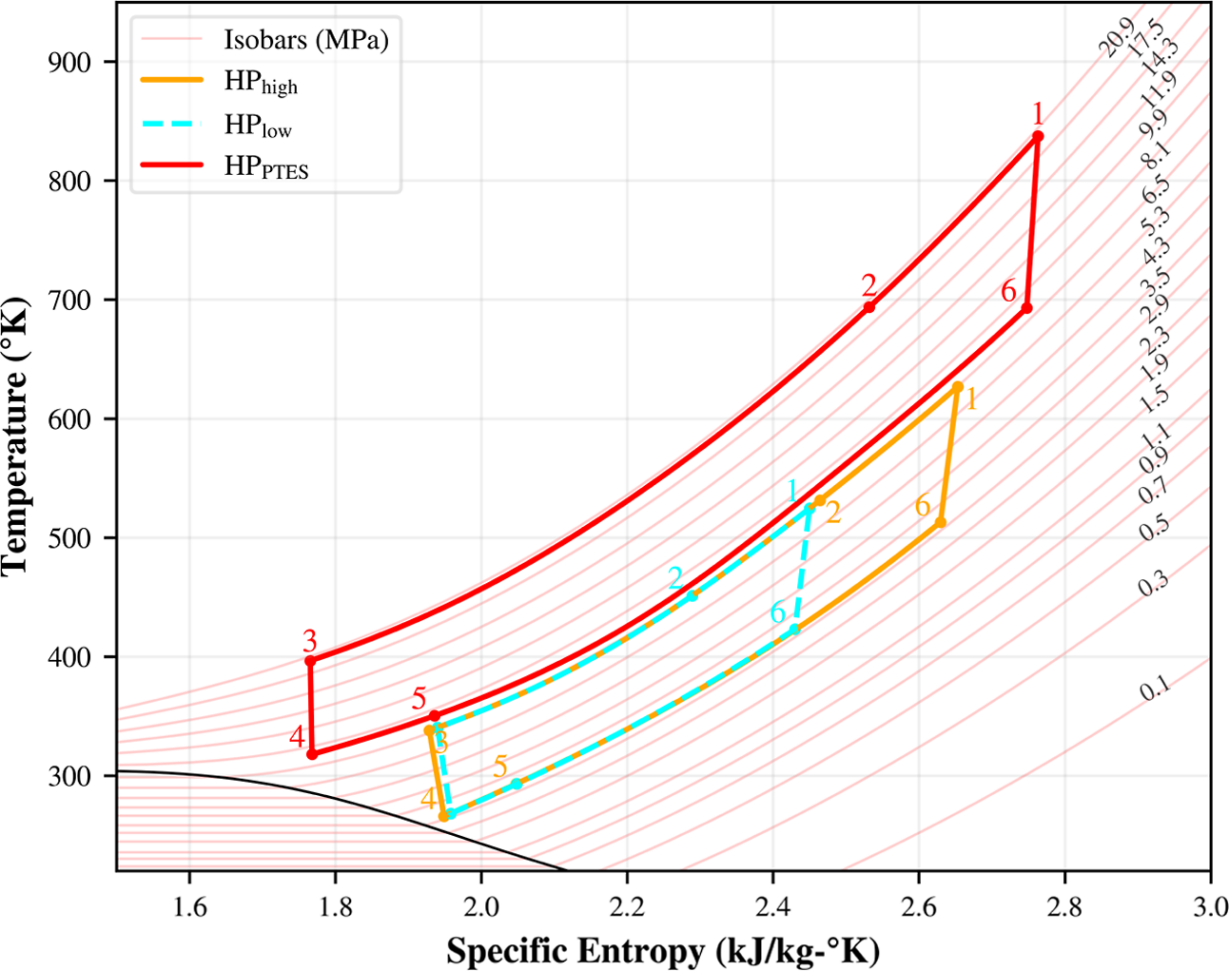
T-s diagram of design point cycle and alternate cycles proposed in.^15^

### 2.2 Storage Material Selection

The critical temperature of the WF determines the cold storage temperature, providing a lower bound to it during charging or an upper bound during discharging, depending on which cycle is designed first. This is based on previous research from the authors^12^ where it was found that to be able to leverage the high compressibility around the critical point during discharging, the CO_2_ exiting the cold storage heat exchanger needs to be as close to the critical point as possible. With the cold storage fixed, the hot storage can be chosen to fit the desired storage temperatures and capacities. However, while the efficiency of the power cycle increases with increasing Δ*T*, the coefficient of performance (COP) of the heat pump decreases with increasing Δ*T* with both effecting the round-trip efficiency (RTE) of the storage system. So, the storage materials need to be chosen based on these principles.

There are multiple options for storage reservoir designs. Storage methods can be classified as either (1) sensible heat storage and (2) latent heat storage.^31^ Latent heat storage relies on phase change, giving ∼50–100× higher energy density than sensible storage and enabling more compact systems. Ice-based cold storage has been studied (Morandin et al.;^24^ Echogen^25^), but performance can be limited by low effective thermal conductivity^33^ as the phase change material (PCM) forms an insulating layer, requiring larger heat-transfer areas. Suitable low-temperature PCMs are typically organics (paraffins/fatty acids/esters)^32^ while high-temperature options are often eutectic salts or hydrides.^33^ Water can be used as ice or steam, though steam storage is costlier due to higher pressure tanks. While phase-change materials can be attractive for their compactness, latent heat storage is around an order of magnitude more expensive compared to sensible heat storages.^34^ However, it is important to note that when considering these TES systems in conjunction with heat intensive industrial processes, the type of required heat is also a factor to be taken into consideration. For instance, if a process requires evaporation or condensation, then a latent heat storage system may become more desirable. Although, in that case it would be an additional challenge to find a storage material that changes phase at the desired temperatures of the process. This highlights the limitation of temperature and pressure dependence of latent heat storage systems. For these reasons, the system considered in this study uses a sensible heat storage layout.

High-temperature sensible storage can be realized using either liquids or solids. Liquid sensible storage is typically implemented as a two-tank arrangement, which is straightforward to operate and provides stable outlet conditions. For the cold side when near ambient temperature, water is an attractive medium due to its low cost and benign safety aspects. For the hot side, molten salts are a mature option with extensive deployment in CSP plants,^35^ offering relatively high storage density at low material cost. Gonzalez-Roubaud et al.^36^ reported that, for storage durations more than 3 h, molten-salt storage can be more viable than pressurized steam storage. Practical constraints include freeze risk at low temperatures, upper-temperature limits associated with thermal stability, and corrosion issues for tanks, piping, and heat exchangers.

Solid sensible storage, such as packed beds,^37^ concrete blocks,^38^ or ceramic media,^39^ has also been proposed for PTES applications. These concepts can tolerate very high temperatures and, in direct-contact configurations, may reduce heat-exchanger requirements by allowing the WF to exchange heat directly with the storage medium. However, for high-pressure Brayton systems, direct contact necessitates pressurized reservoirs. In addition, thermocline formation leads to time-varying outlet temperatures, which can reduce effective utilization and impose tighter control requirements to maintain acceptable turbomachinery inlet conditions.^40^ McTigue et al.^40^ showed that deeper cycling can mitigate thermocline effects; however, long-duration storage operated against variable demand may not always achieve the required depth of cycling. Additionally, solid storage typically offers fewer independent control variables than liquid storage, since the storage medium is stationary and cannot be actively circulated to shape the thermal response.

For these reasons, and to aim for simplicity and industrial applicability, this study adopts liquid sensible storage. Since the design targets multitemperature heat delivery, multiple storage temperature bands are required. The reference system forming the basis of this work utilized molten salt storage between 420–560 °C.^12^ The subsequent multilevel extension^15^ used Therminol 55/66 for the additional MHP and LHP hot storages, selected for their suitability within the targeted temperature ranges. Together, these storage stages enable heat supply over a range of 120–560 °C.

On the cold side, the HHP system uses warm water between 35 and 90 °C.^12^ The MHP and LHP utilize an ambient temperature heat source, which can be either air or water. Both in^15^ and this work, the goal is to use the same equipment for HHP, MHP and LHP so that the capability of a single PTES layout delivering heat at multiple temperatures can be assessed. Since the CHX in the main heat pump (HHP) is designed for CO_2_-water heat exchange, water is used in MHP and LHP as well, albeit at lower temperatures. This reduces the complexity associated with changing thermodynamic cycles as the only additional equipment needed for the heat exchangers would be valves and additional piping.

### 2.3 Operational Strategy

The operational strategy of the system is shown in Figure 5. In previous work^12,15^ a state-of-charge (SoC) management-based dispatch strategy was developed. The optimization algorithm used a surrogate model of the PTES system developed in ASPEN and electricity price data to maximize operational profit over a given simulation horizon. This results in a charge–discharge profile shown in Figure 6. The optimization algorithm satisfies a constant demand of heat at three different levels as well as operating profitably providing grid services.

**Figure 5:**
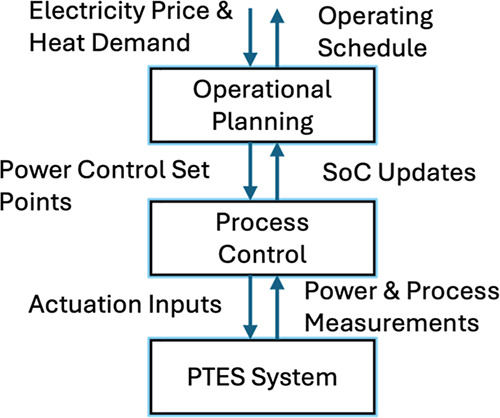
Proposed operational strategy.

**Figure 6:**
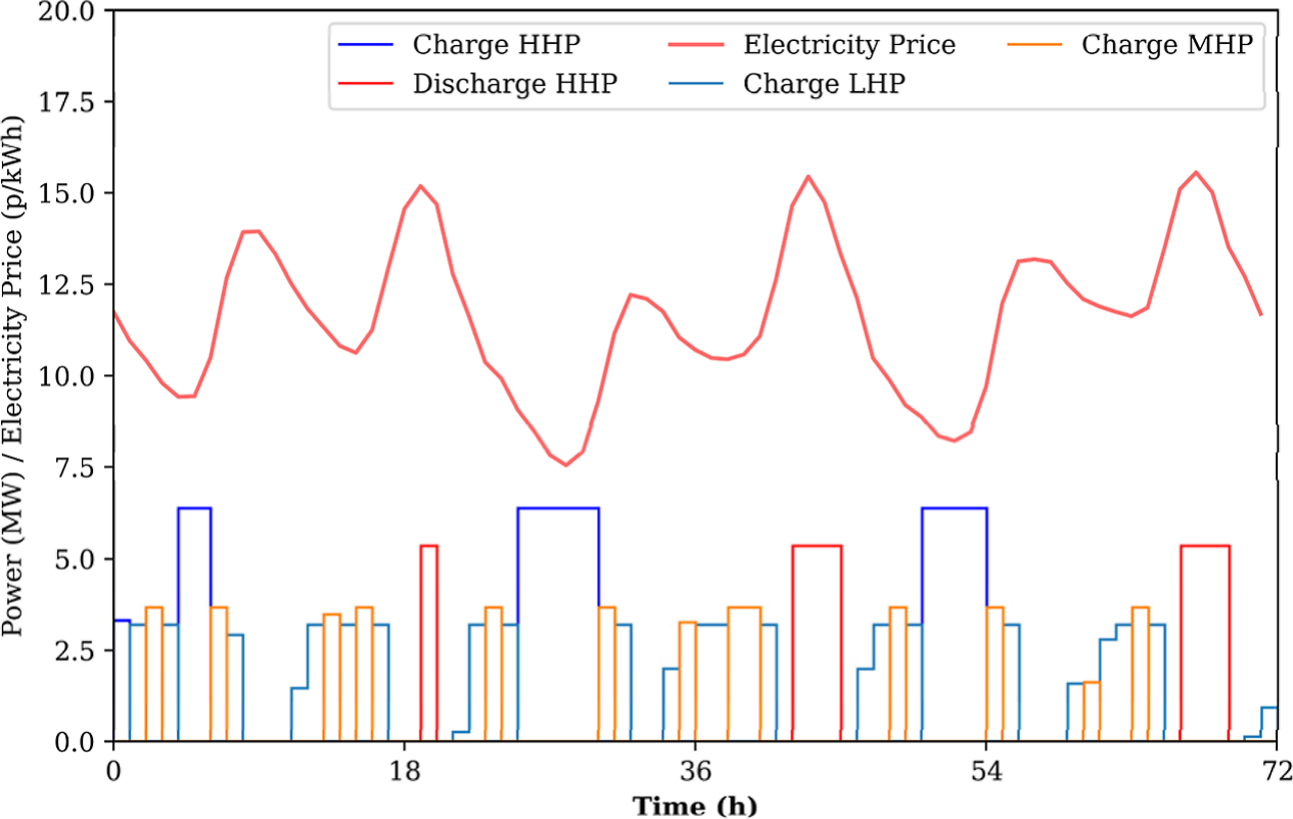
Sample operational schedule from scheduling optimization study, adapted from.^15^

However, as evident in the operating schedule in Figure 6, there are rapid changes in the operating point of the PTES system. For an accurate implementation of such a system a transient analysis is necessary. Initially, understanding how such a system can be controlled is imperative. Then, the resulting understanding of the transient behavior can improve and inform the scheduling optimization studies by introducing ramp rate constraints between operating points.

## 3 Methodology

### 3.1 Dynamic Modeling

Accurately capturing transient behavior is essential for regulating power output and integrating supercritical CO_2_ cycles with real-time grid load fluctuations. To understand this transient behavior of PTES systems under off-design conditions, it is imperative to build a dynamic model. This study used MATLAB Simulink.^41,42^ Three submodel types were developed: a compressor model, heat-exchanger models, and a turbine/expander model. Notably, the heat exchangers differed from each other slightly as the different fluids required different calculation strategies. These differences are explained in Section 3.1.2. The model used REFPROP^43^ for fluid properties.

The discharging phase of PTES systems has received greater research attention and studies such as those by Yang et al.,^44^ and Wang et al.,^45^ developed detailed dynamic models of Brayton-based PTES systems to capture compressor–turbine inertia and transient instabilities. Together, these works show how PTES studies have evolved from established power cycle research, positioning the dynamic behavior of the discharging phase as a key focus for efficiency and grid flexibility. On the heat pump side, Oehler et al.^46^ explored the startup of the CoBra test rig of the German Aerospace Center (DLR), which utilizes an air-based Brayton cycle. Tran and Stathopoulos’ work^47^ built a dynamic model of the CoBra rig and validated it with data from the rig. Pettinari et al.^48^ studied the part-load behavior of a Brayton Heat Pump in a dynamic setting using data from the same DLR rig.

On complete PTES systems, Xue and Zhao^49^ looked into transient behavior of packed bed latent heat storage. Further research by Lu et al.^20^ and An et al.^22^ explored startup effects and Frate et al.^50^ studied different control strategies necessary in PTES systems.

The identified research gaps in this space are (1) there are more studies about the dynamic behavior of the power cycle than the heat pump cycle. The recent work done on the heat pump cycle was based on the DLR test rig, meaning the studies were based on air cycles.^46,47^ The holistic PTES work by Frate et al.^50^ focused on Argon cycles so there is an opportunity to fill in gaps for sCO_2_ Brayton cycle research. (2) There is currently limited research on multitemperature heat pumps from a single source, especially regarding transients when changing the cycle pressures and temperatures. So, this study will focus on the heat pump cycle of a supercritical CO_2_ Brayton Cycle under changing cycle conditions and its transients.

#### 3.1.1 Compressor Model

The compressor was modeled using the method developed by Gravdahl and Egeland^51^ and summarized by Cortinovis et al.^52^ The model consists of the following equations.

1 $$\frac{d\omega }{dt}=\frac{1}{J}\left(\tau_{drive}-\tau_{comp}\right)$$

2 $$\frac{d\pi_{c}}{dt}=\frac{1}{t_{\pi }}\left(\pi_{c,ss}-\pi_{c}\right)$$

3 $$\frac{d{ṁ}_{comp}}{dt}=\frac{A_{c}}{L_{c}}\left(p_{1}\cdot \pi_{c}-p_{2}\right)$$

4 $$\frac{d{ṁ}_{rec}}{dt}=\frac{1}{t_{rec}}\left({ṁ}_{rec,ss}-{ṁ}_{rec}\right)$$

5 $$\frac{dP_{1}}{dt}=\frac{a_{1}^{2}}{V_{1}}\left({ṁ}_{in}+{ṁ}_{rec}-{ṁ}_{comp}\right)$$

6 $$\frac{dP_{2}}{dt}=\frac{a_{2}^{2}}{V_{2}}\left({ṁ}_{comp}-{ṁ}_{out}-{ṁ}_{rec}\right)$$

7 $$\pi_{c,ss}=f\left({ṁ}_{comp},\omega \right)$$

8 $$\tau_{comp}=\sigma \cdot r^{2}\cdot \left|{ṁ}_{comp}\right|\cdot \omega$$

9 $${ṁ}_{in}=C_{v_{in}}z_{in}\sqrt{P_{L}-P_{1}}$$

10 $${ṁ}_{out}=C_{v_{out}}z_{out}\sqrt{P_{2}-P_{H}}$$

11 $${ṁ}_{rec,ss}=C_{v_{rec}}z_{rec}\sqrt{P_{2}-P_{1}}$$

The dynamic states are the shaft speed ω, compressor pressure ratio π_c_, compressor mass flow *m*_comp_, recycle-line mass flow *m*_rec_, and the inlet/outlet plenum pressures *P*_1_ and *P*_2_ (see Figure CompDia for the sign convention). The shaft dynamics (eq 1) are given by a torque balance with inertia *J*, where τ_drive_ is the applied drive torque (input) and τ_comp_ is the aerodynamic load torque computed from eq 8. The pressure ratio dynamics (eq 2) are modeled as a first-order lag toward the quasi-steady compressor map value π_c,ss_, from eq 7, with time constant *t*_π_. Equation 3 represents the mass flow through the compressor volume using the pressure difference between the inlet (P1) and the outlet (P2) plenum. Equation 4 models the recycle mass flow rate. The inlet, outlet, and recycle valves shown in eqs 9–11 determine *m*_in_, *m*_out_, and *m*_rec_ through the valve relations. The inlet and outlet plenum pressures are obtained from mass conservation in each plenum (eqs 5 and 6), where *a*_1_ and *a*_2_ denote the local speed of sound in the inlet and outlet plenums, respectively. Relevant variables and their associated components are shown in Figure 7.

**Figure 7:**
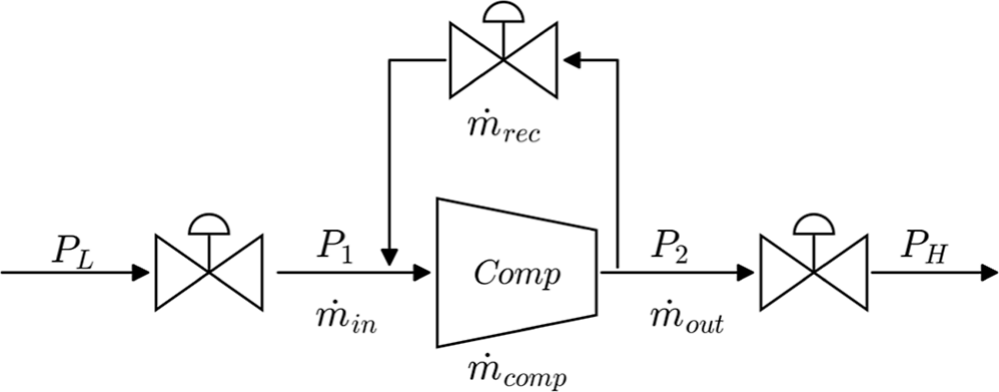
Compressor model components and variables.

*P*_L_ and *P*_H_, the low and high pressures of the cycle respectively, are similarly calculated using conservation of mass in the cycle in general.

12 $$\frac{dP_{L}}{dt}=\frac{a_{L}^{2}}{V_{L}}\left({ṁ}_{cycle}-{ṁ}_{in}-{ṁ}_{vent}\right)$$

13 $$\frac{dP_{H}}{dt}=\frac{a_{H}^{2}}{V_{H}}\left({ṁ}_{out}-{ṁ}_{vent}-{ṁ}_{cycle}\right)$$

Where subscripts L and H denote low- and high-pressure sections. *V*_L_ and *V*_H_ are the corresponding volumes, and *a*_L_ and *a*_H_ are the local speeds of sound (computed from the section thermodynamic state). Here ${ṁ}_{cycle}$ is the mass flow through the expander (positive from high to low pressure). The vent flows ${ṁ}_{vent,L}$ and ${ṁ}_{vent,H}$ are controlled by dedicated valves and an auxiliary compressor, as described in the section on inventory control.

#### 3.1.2 Heat Exchanger Model

The heat exchanger models are modeled in 1D-finite volume models, where the full volume of the heat exchanger is divided into N segments. Each segment is assumed to be well mixed. The number of segments differed for each heat exchanger, depending on the complexity of the internal heat exchange behavior. Figure HEX is a diagram of the model. The general form of the modeling equations is as follows

14 $$\rho_{i}V_{i}\frac{dh_{i}}{dt}=ṁ\left(h_{i-1}-h_{i}\right)-U_{i}A_{i}\left(T_{i,hot}-T_{i,cold}\right)$$

where *i* is the index of the segments in the heat exchanger. The term *h*_*i*_ is the bulk specific enthalpy of the fluid in segment *i*. The factors ρ_*i*_, *V*_*i*_ and *A*_*i*_ are the density, volume and area of the control volume. *ṁ* is the mass flow rate of the fluid, and *T*_*i*,hot_ and *T*_*i*,cold_ are the local bulk temperatures of the hot and cold streams in segment *i*.

*U*_*i*_ was calculated using the heat transfer coefficient correlations suggested by Baik et al.^53^ for PCHEs, assuming semicircular channels.

15 $${Nu}_{water}=0.2829{Re}^{0.6686}$$

16 $${Nu}_{{CO}_{2}}=0.8405{Re}^{0.5704}{\mathrm{Pr}}^{1.08}$$

Three of the heat exchangers, the cooler, CHX, and REC were modeled using these forms of the differential equations and heat transfer correlations. However, the salt or the Therminols used in the hot storage is not available in REFPROP. Their property information was obtained from separate sources,^54,55^ which did not include a correlation for specific enthalpy. So, for the hot side, the following differential equation was used

17 $$\rho_{i}V_{i}C_{p,i}\frac{dT_{i}}{dt}=ṁC_{p,i}\left(T_{i-1}-T_{i}\right)-U_{i}A_{i}\left(T_{i,hot}-T_{i,cold}\right)$$

where *C*_p,*i*_ is calculated from REFPROP or correlations for molten salt and Therminols.

First, the heat exchangers were sized by simulating the design conditions. Initially, total area is calculated, to meet the design outlet temperatures. Then, the volume of PCHEs were estimated. According to Hesselgraves^56^ and Oh et al.,^57^ the heat transfer surface area density can estimated as 2500 m^2^/m^3^. This value was used to calculate the volume of the heat exchangers individually. In subsequent simulations, the volume and area were fixed to these calculations.

#### 3.1.3 Expander

The turbine/expander is modeled as an isentropic expansion from *P*_H_ to *P*_L_, both provided as an input to the model, with a simple efficiency-based power correlation and first-order output dynamics. The expander model receives η as an input from the compressor model. Turbine power is computed using an ideal-gas isentropic relation with κand inlet *C*_p_ from a REFPROP

18 $$Ẇ=ṁC_{p}T_{in}\eta \left(\Pi^{-\left(\kappa -1\right)/\kappa }-1\right),\Pi =P_{out}/P_{in}$$

At very low flow rates (during startup/shutdown) the specific work is set to zero for numerical robustness. The outlet temperature used in the model is obtained by converting the extracted specific work to an enthalpy drop and calling REFPROP for *T*(*P*,*h*). Finally, mass flow, outlet temperature, and power are each passed through first-order lags, $ẏ=K\left(y^{★}-y\right)$ , to represent component and control dynamics.

Each model is connected as shown in Figure 8. As mentioned, the speed and power controller are used in different operating modes. Some lines that made the diagram too crowded were represented as to/from tags, such as the idle signal from the compressor to the heat exchangers. Although the figure shows the layout of the heat exchanger controllers as controlling storage temperatures, they can be wired to control CO_2_ outlet temperatures during cycle design.

**Figure 8:**
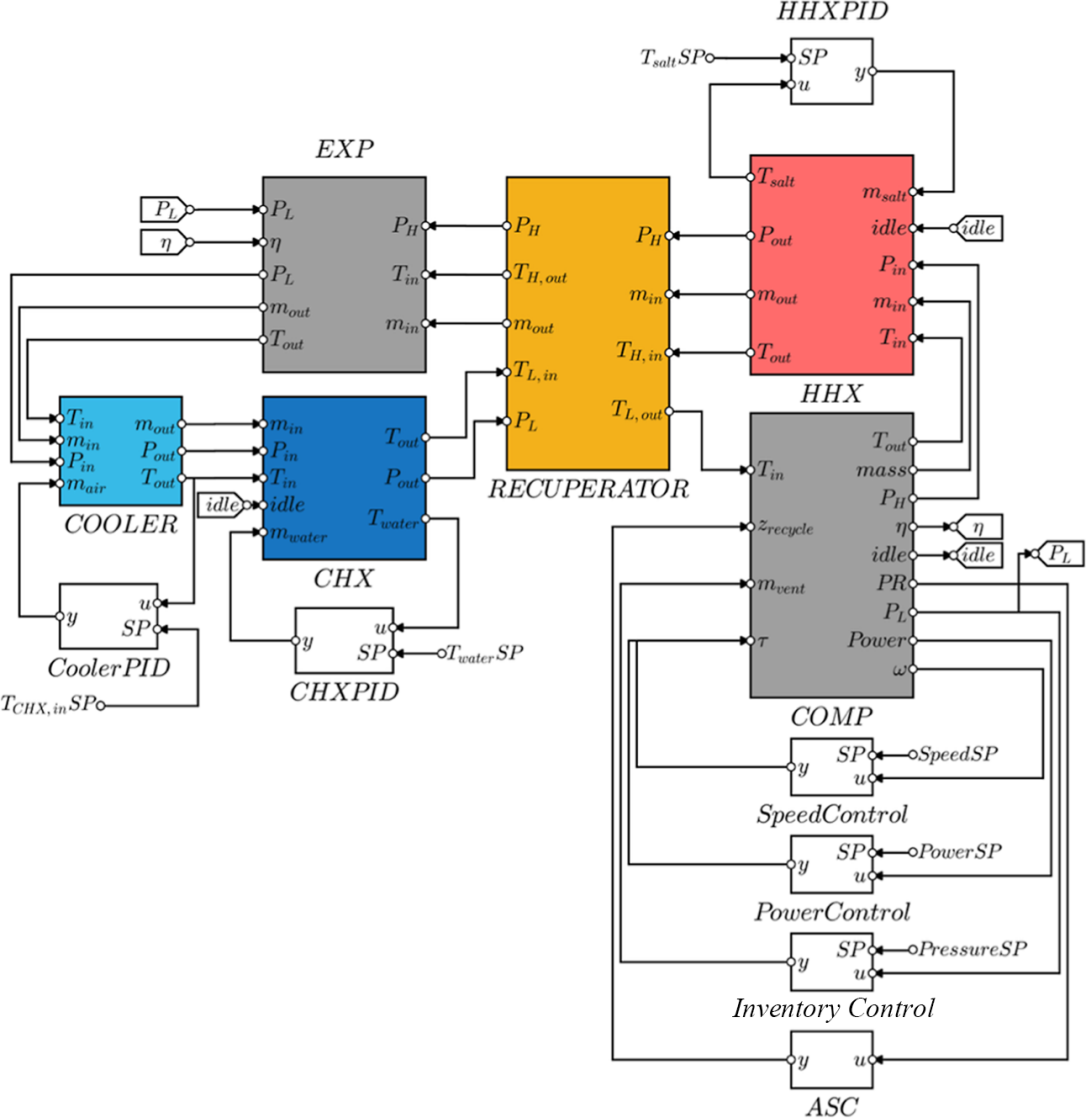
Connections of the PTES system submodels and controllers.

### 3.2 Fluid Properties

Direct REFPROP calls were a computational bottleneck: with more than 30 segments per heat exchanger, repeated calls significantly slowed the simulation. Therefore, REFPROP was used to generate property lookup tables offline, which the simulation used to interpolate property values faster. The performance of the lookup tables was assessed in smaller simulations in comparison with REFPROP and was deemed adequate. It would be outside the scope of this work to outline the results. The authors can provide code and performance metrics on request.

### 3.3 Compressor Map

The compressor behavior was captured with a compressor map, input to the model as a Gaussian Process. It is the same compressor map from.^12,14,15^ The GP takes mass flow (ṁ) and rotation speed (ω) as inputs and outputs pressure ratio (π_c_) and efficiency (η).

### 3.4 Control Objectives

Figure 9 shows the layout of the controllers and the actuators on the PTES heat pump process flow diagram. There are several control objectives pertinent to the PTES system. These can be broadly classified into two categories, safety and performance.

**Figure 9:**
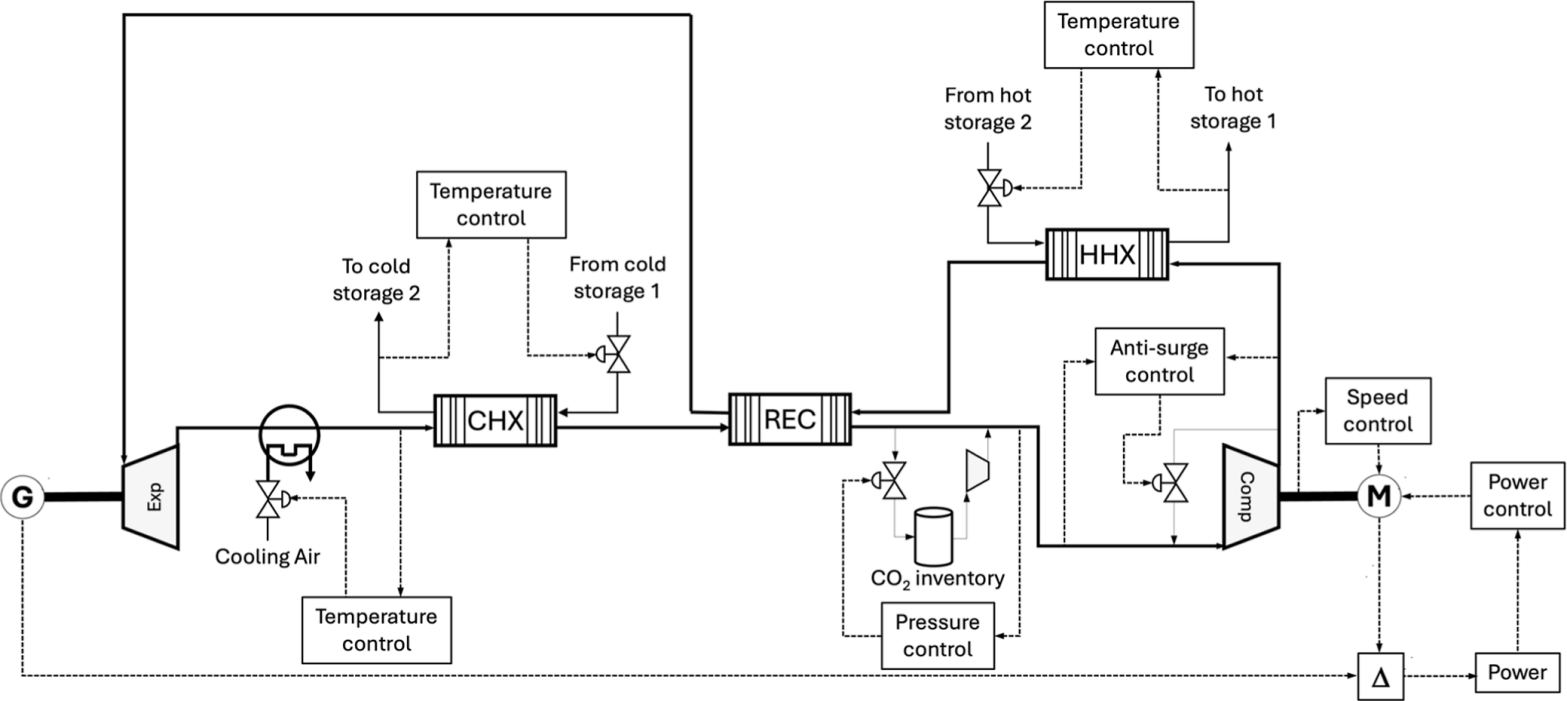
Plantwide control schematic of the PTES heat pump.

The main safety objective is the safe operation of the turbomachinery which is accomplished by antisurge control (ASC). Surge occurs at low compressor flow rates and causes fluctuations in flow and pressure that leads to adverse performance effects for the compressor. These events, if not controlled, may damage compressor components. The goal of ASC is to keep the operating point of the compressor below the operating line known as the surge limit. This surge line seen in Figure 10, provides a relationship between flow rate and pressure ratio. For a given compressor shaft speed, it signifies the lower limit on flow. Main path of ASC involves controlling the opening of a valve to either vent or recycle the exhaust gas of the compressor.^52^ In this study, the physical method developed by Cortinovis et al. is used. A so-called surge margin is tracked throughout the operation, which measures the distance of the operating point from the surge line. This is used to control the recycle valve opening to maintain safe operation and distance from the surge line. As the resistance of the upstream processes change, such as a blockage in a pipe or a valve closing, the resistance curve becomes more vertical. For the same shaft speed, as the curve changes, the operating point also changes and moves closer to the surge line. Opening the recycle valve essentially reduces the resistance, restoring the operating point. However, during recycle, work imparted onto the recycled gas is wasted so it is inefficient to always keep the recycle valve open.

**Figure 10:**
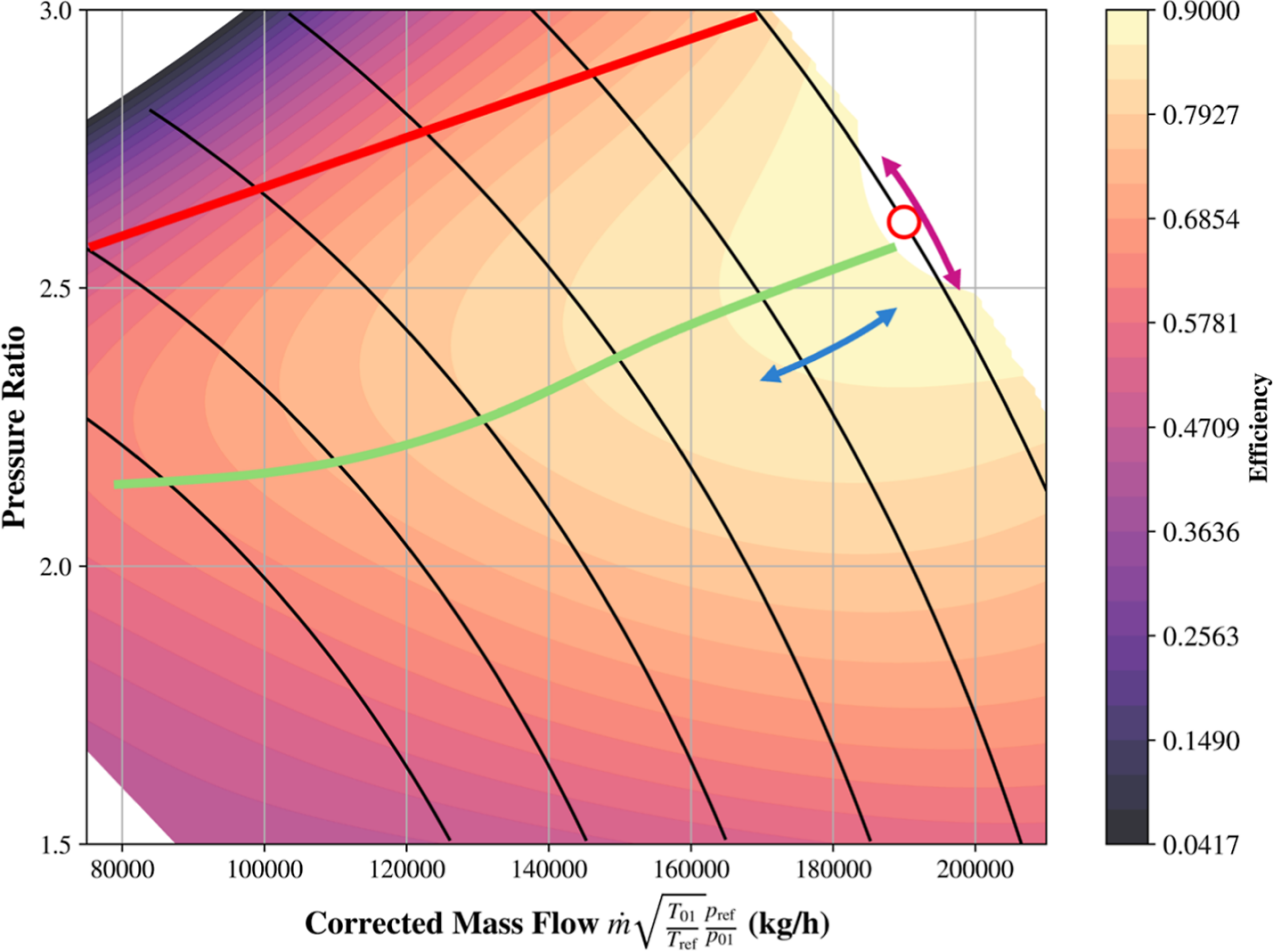
Sample compressor map. Shown are surge line (red), resistance curve (green), direction of inventory control (purple), direction of speed control (blue) and the design point (red circle).

Another safety objective is maintaining the heat storage temperatures and the compressor inlet conditions within the operating limits. This is mainly done in the 3 external heat exchangers, by controlling the hot storage (molten salt or Therminol), cold water and cooling air flow rates. For instance, the molten salt should always be kept above its freezing point. Similarly, the Therminol storages should be kept below its degradation point. In fact, temperature controls play a part in both operational and safety control.

Controlling the performance of the heat pump involves manipulations to change its operating point to satisfy operational objectives. This involves power control and inventory control. Variable speed compressors allow for power control by changing the rotational speed and let the operating point glide along the resistance curve of the cycle, shown in orange in Figure 10. This in turn changes the mass flow rate through the compressor and thus the operating power.

The system tracks the power of the system and manipulates the input torque of the shaft. While this control regime can be used when the system is tracking a power set point, it is not accurate during shutdown, startup and cycle change. Instead, since the power of the system is not relevant when shutting down the speed is controlled directly from the torque. The speed is set to 0 during shutdown and the nominal operating speed at startup. Also, below a certain lower bound of shaft speed, the compressor map becomes inaccurate. It is necessary to exit the curve at that point and fully open the recycle valve so that the compressor can spool up or down without any detrimental effects. So, in this low-speed region, in addition to shaft speed control, recycle valve is also controlled.

Inventory control, applied to PTES systems by Frate et al.,^50^ allows controlling compression cycle operating conditions, when fixed speed turbomachinery is used. This strategy changes the holdup of the WF in the cycle by venting or injecting it to and from external storage tanks. This changes the pressure and the resistance curve of the cycle. In fixed speed machines, this is the only option for controlling the system power and operating point. However, variable speed machines provide an additional degree of freedom. By changing the content of the WF in the cycle, the lower temperature cycles of the MHP and LHP can be reached.

## 4 Results

This section contains the results of the dynamic control study of the PTES system. All settling times are reported in the form *t*_s,2%_, settling time to within 2% error band of the final value *P*_f_ where *P*_f_ is the average value of the last 100 s of the given data. In each case, the reported time is relative to the set point change, which is different for each study and reported where appropriate.

All of the controllers used in this study are continuous PI controllers. The controllers were tuned by simulating medium size random disturbances and minimizing settling time and steady state error. Specific settings are provided in relevant sections.

### 4.1 Anti-Surge Control (ASC)

Anti-surge control was achieved by defining a surge margin line on the pressure ratio-mass flow space of the compressor map. The upper end of the compressor speed lines seen in Figure 11 is determined by the available data that was used to generate the compressor maps. Beyond that line, the map is inaccurate and it was chosen as the surge line. Based on the surge line, a surge margin was determined, shown in yellow, so that there is on average, 12% error between the surge margin and the surge line. Since below the surge margin the controller would be saturated, an antiwindup scheme was used. The error calculated for the controller was between the current operating point and the point of the surge margin on the current speed line. When the error was below 0, the controller output was clamped and no integrator error was registered.

**Figure 11:**
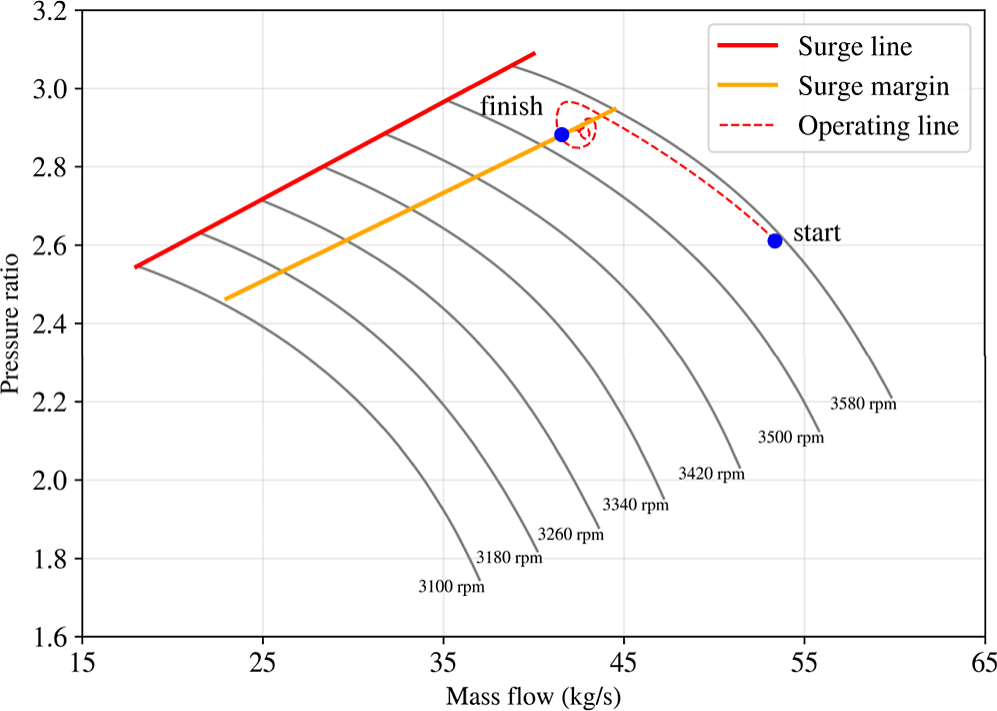
Compressor speed lines (black), operating line and surge limit and surge margin lines.

To demonstrate the response and effectiveness of the anti-surge control (ASC) system, the discharge of the compressor was restricted by ramping the compressor outlet valve from fully open to 20% open over 75 s (Figure 12, top). This throttling reduced the compressor mass flow while increasing the pressure ratio, causing the operating point to migrate along the speed line toward the surge boundary (Figure 11). During the early part of the disturbance, the controller remains inactive because the operating point lies beyond the surge margin; consequently, the pressure ratio continues to rise and the mass flow continues to fall as the outlet valve closes (Figure 12, bottom). Once the trajectory approaches the surge line and the surge margin diminishes, the ASC rapidly opens the recycle valve to relieve the developed pressure rise (Figure 12, top). The aggressive initial corrective action produces a brief overshoot and short-lived oscillations in both pressure ratio and mass flow (Figure 12, bottom), after which response damps out quickly. The system then converges to a stable operating condition that lies on (or very near) the surge boundary for the active shaft speed, as indicated by the “finish” point on the compressor map (Figure 11). Overall, the combined map-based trajectory (Figure 11) and time-domain response (Figure 12) show that the ASC activates only when required, restricts movement into the unstable region for the compressor, and stabilizes the compressor at the limiting boundary with minimal settling time.

**Figure 12:**
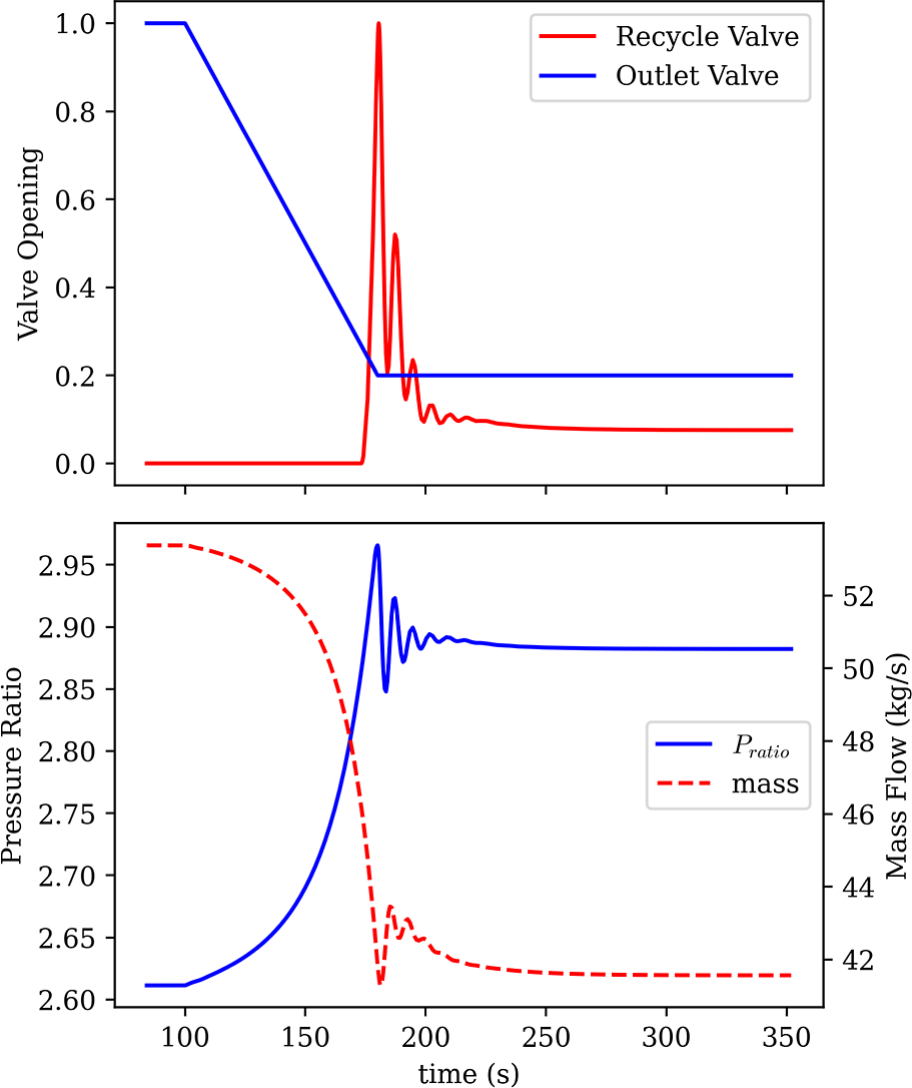
ASC Performance. Top: disturbance profile and recycle valve opening. Bottom: controlled variables.

### 4.2 Power Control

The power controller and the system’s power tracking capability were assessed by applying a set of negative power steps from the design power of 9.6MW (compressor power) spanning from −0.4 MW to −4.0 MW in equal 0.4 MW increments, starting at *t* = 50. The set point changes and the system response is shown in Figure 13a. The controller achieved *t*_s,2%_, between 15 and 45s, increasing with step magnitude. Figure 13b shows the change in the cycle T-s diagram from the design point to the minimum power case simulated.

**Figure 13:**
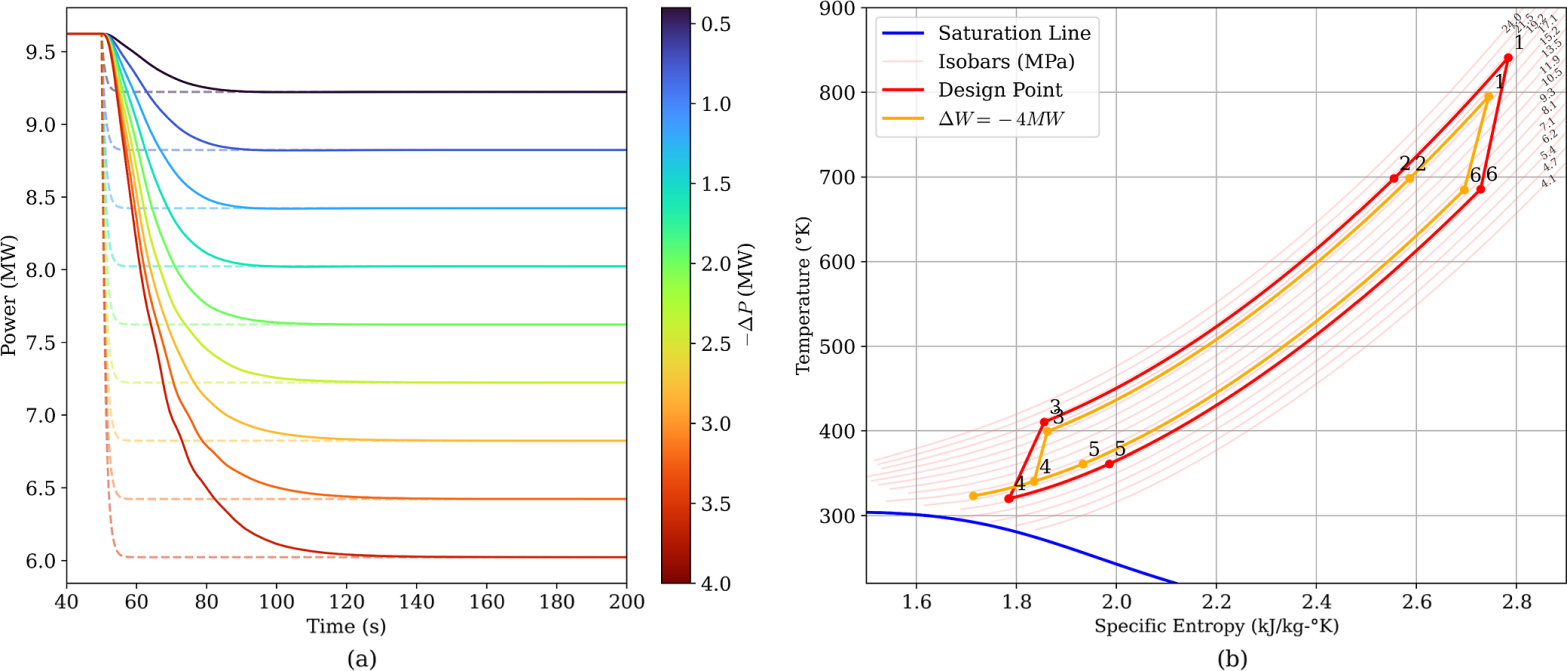
(a) Power step changes and system response. (b) T-s diagram of the design point and the minimum power simulated.

The corresponding temperature responses in Figure 14 show how the temperature controls accommodate the power reduction across the cycle. The compressor outlet temperature decreases as the compressor work is reduced, consistent with the reduction in the compressor inlet temperature, i.e. recuperator cold outlet. Both recuperator outlet temperatures shift to a lower steady level consistent with the reduced cycle temperature lift. The cooler outlet and CHX outlet primarily show transient deviations during the power change before returning close to their prestep values. The small oscillations visible on the CHX outlet temperature trace are likely numerical (e.g., solver tolerance/step-size effects or discretization artifacts) rather than a physically meaningful thermal instability. Overall, Figure 13a demonstrates stable power tracking over the full range of step magnitudes, while Figure 14 indicates that the associated thermal excursions remain bounded and settle to consistent new operating conditions.

**Figure 14:**
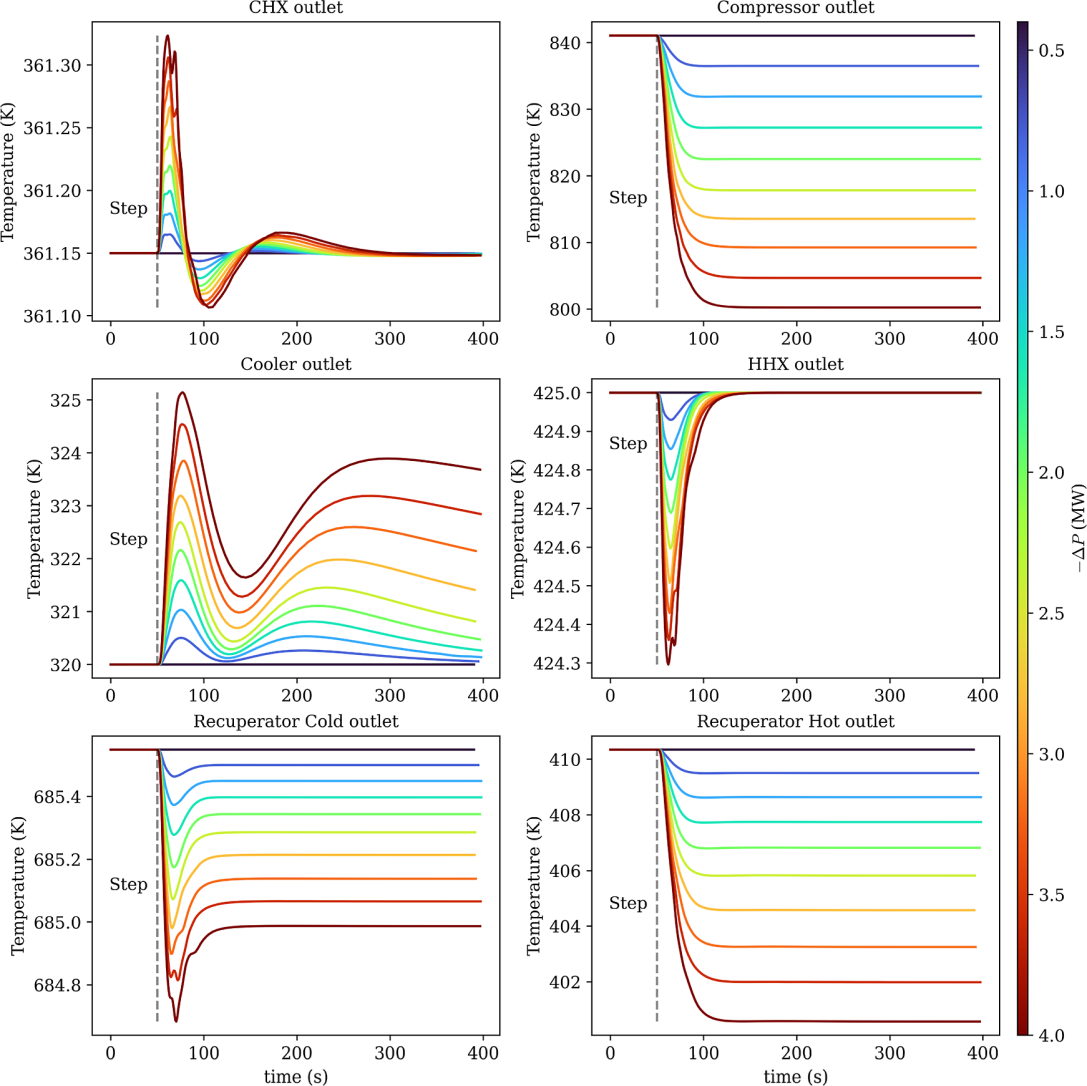
Temperature response during power tracking.

### 4.3 Inventory Control & Operating Point Change

To accommodate multiple supply temperatures, the system needs to operate at different cycles. While the HHP is designed to operate at a low cycle pressure of 7.4 MPa, and a high pressure of 18.52 MPa and between 46 and 567 °C. Whereas the MHP cycle operates between 2.3 and 5.4 MPa and −13 and 416 °C. The simulation in this section is only for HHP-MHP switching; however, it is a representative model of other temperature switches that can be accommodated by this method. Figure 15 shows the T-s diagrams of the two cycles. The control scheme involves first changing the pressure set point to the new operating pressure. This occurs at *t* = 50s. Then, 200s later, the temperature set points are changed to the new values. Table 1 shows the set points of the two cycles. To facilitate these changes, the water, Therminol and coolant air supply temperature are also adjusted. In HHP, water is supplied to the system from the storage tanks at 90 °C, whereas in this layout, since the water will not be stored, it is supplied at ambient temperatures (in this case assumed to be 21 °C, but could be higher if utilizing waste heat from a coupled process) and is cooled as much as possible without freezing. Similarly, the hot storage inlet temperature is changed from 420 °C for molten salt, to 295 °C for Therminol. The cooling air, which is only used during the transient between the two cycles is supplied at −20 °C.

**Figure 15:**
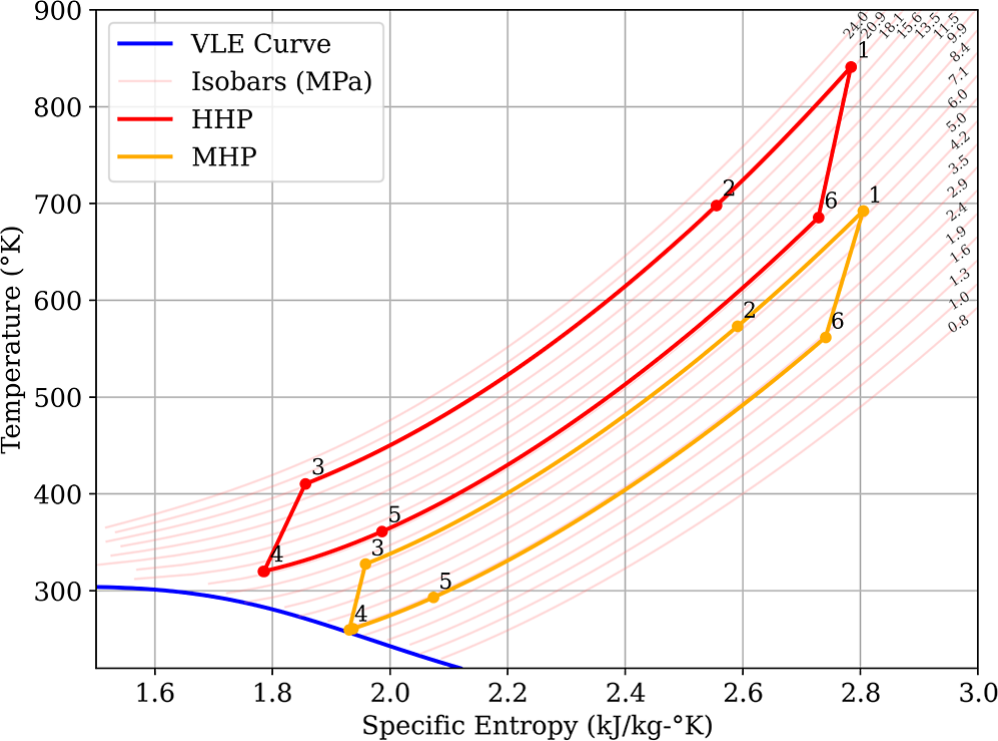
T-s diagram of HHP and MHP cycles.

**Table 1:** Cycle Change Set Points

	HHP	MHP
cycle pressures (MPa)		
*P*_low_	7.4	2.3
*P*_high_	18.5	5.4
*P*_ratio_	2.5	2.35
outlet temperatures (°C)		
HHX	425	300
CHX	88	20
Cooler	46	–13

The control inputs used during cycle change were the low-pressure vent and the recycle from the high-pressure side. Figure 16a shows the inventory control mass flows. Here, mass from the low-pressure side is vented to the inventory tank (*m*_vent_ to tank) and mass from the high-pressure side is recycled before the hot heat exchanger back down to the low-pressure side (*m*_recycle_ to low pressure side). This should not be confused with the recycle flow for ASC, which is essentially within the boundary of the compressor and recycles CO_2_ from the outlet plenum to the inlet plenum. Additionally, during the cycle change speed control is used instead of power tracking as the power of the final cycle changes due to the lower inlet conditions.

**Figure 16:**
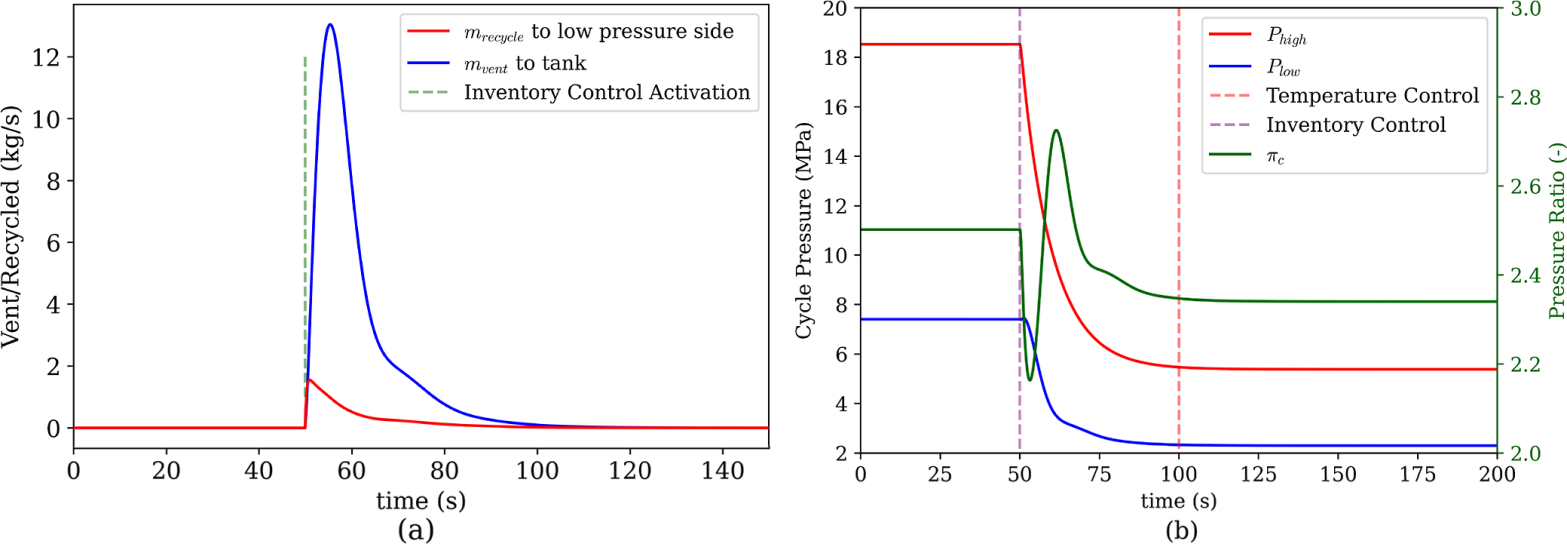
(a) CO_2_ inventory management flow rates. (b) transients of high and low pressures of the system.

As with the other cases, the settling time for the pressures are much faster than the heat exchangers. Both the high and low pressures settle to the new steady state in around 48s. Temperature control activation does not have an impact on the pressure change, as evident from Figure 16b. Since temperature controller switching starts later, there is a period where the old set points are in effect. Indeed, when the cycle pressures start changing, the initial cycle set points can mitigate some of the impact of the changing temperatures. Then, as soon as the new temperature set points take effect, the settling time, *t*_s,2%_, is, on average, 73s (Figure 17). In fact, the slowest response is the cooler’s response, at 133.3s. Without this, the average comes out to be 61s. The sluggish response of the cooler can be better analyzed looking at Figure 18, where the hot storage, water and cooling air flow rates through the transient are shown. The cooler is stabilizing the CHX inlet temperature during the transient behavior and soon after the cycle settles, air flow stops.

**Figure 17:**
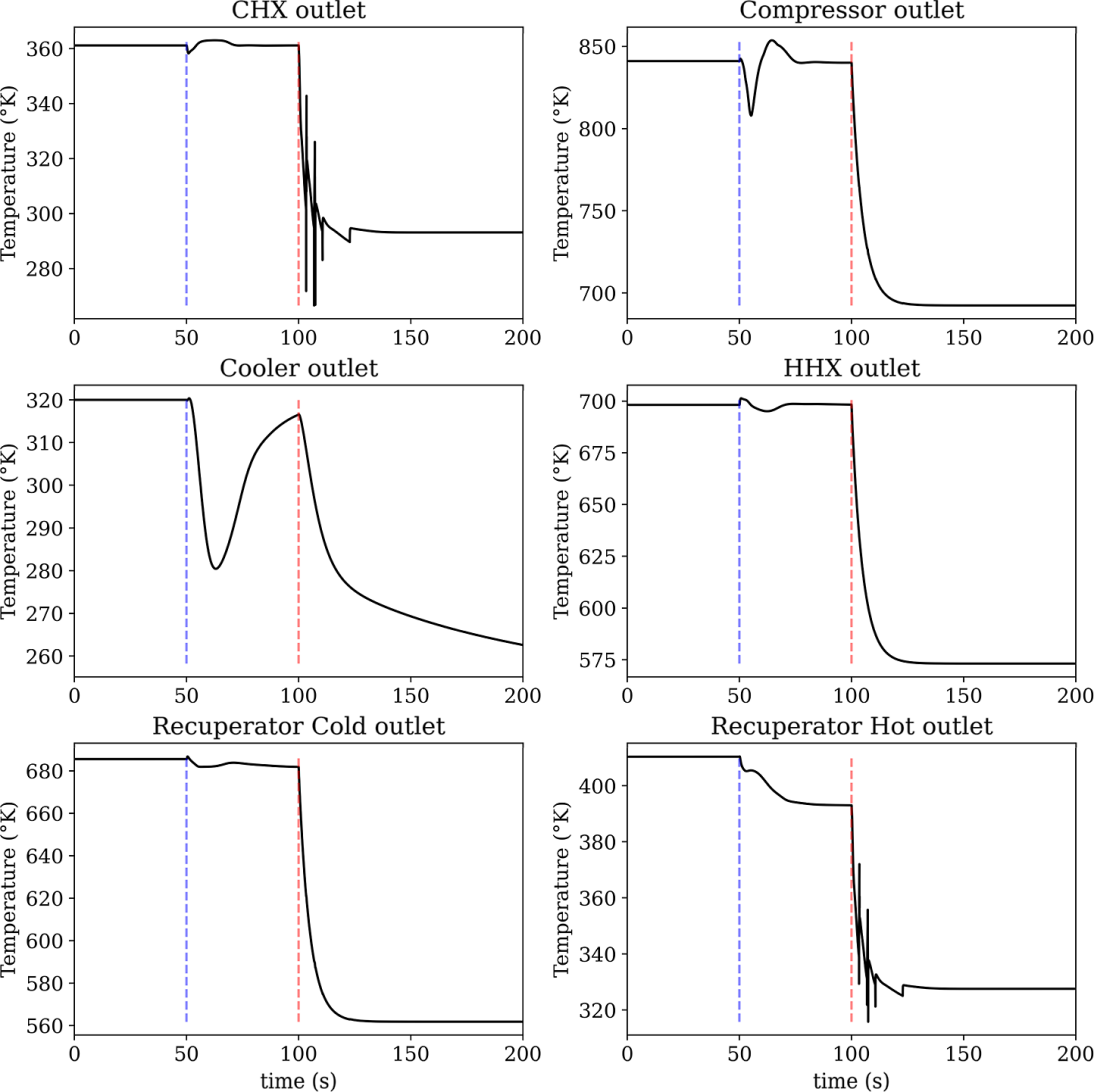
Temperature response during cycle change. Vertical dashed lines: (1) inventory control activation, (2) temperature control activation.

**Figure 18:**
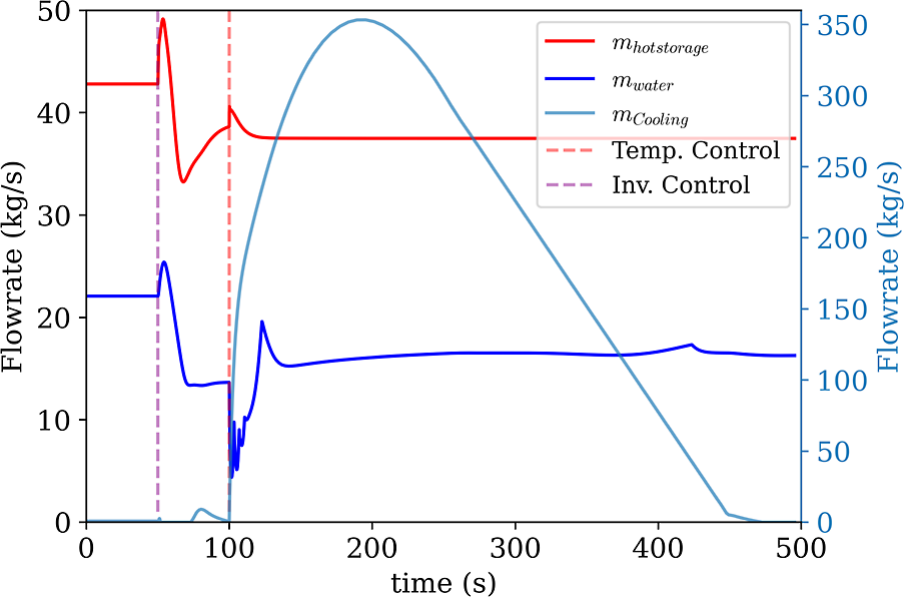
External heat exchanger mass flows. HHX (molten salt or Therminol) and CHX (water) flow rates plotted on the left axis and cooler (air) flow rate on the right axis. Vertical dashed lines: (1) inventory control activation, (2) temperature control activation.

### 4.4 Startup and Shutdown

Startup and shutdown were simulated by changing the power set point to 0 at 100s and again to nominal power 9.6MW, at 1200s. Figure 19 shows the power response. The model uses the compressor map when the shaft speed is above 2000 rpm. However, as the compressor map extrapolation gets more inaccurate at lower speeds, at these speeds, the model uses a linear slope down to 0 rpm. This reduces the system’s erratic behavior at low speeds and ensures a smooth transition from spooling to operational speeds. During shutdown, the system has a settling time (*t*_s,2%_) of 165.4s, and during start-up, it has a settling time of 91.3s.

**Figure 19:**
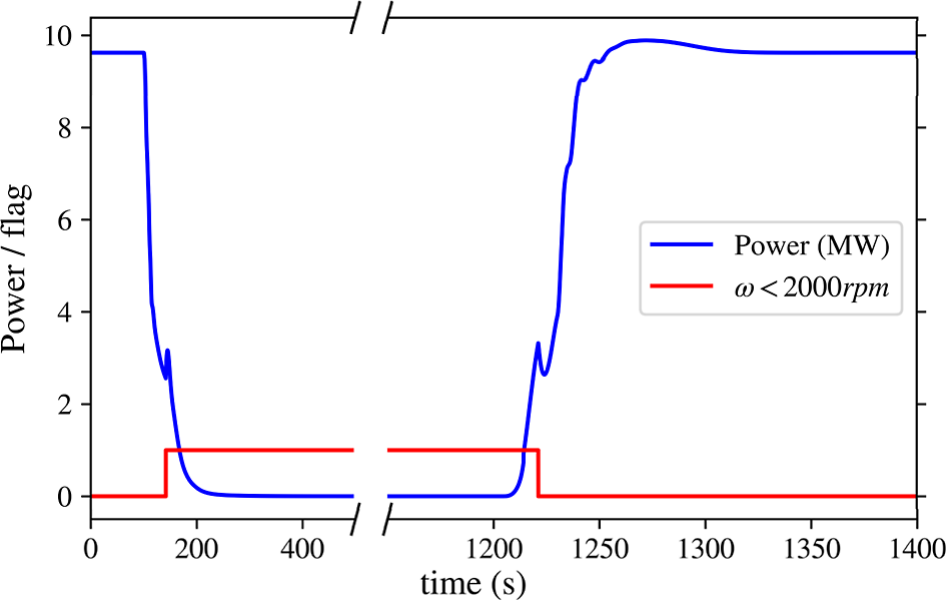
Power change during shutdown (*t* = 50) and startup (*t* = 1200). Also shown is the system flag for when the model is not using the compressor map. Note: Discontinuities at *t* = 160s and *t* = 1220s are simulation artifacts from switching between compressor map and linear extrapolation.

The discontinuities around *t* = 160 and *t* = 1220, are due to the switching between the map and the linear extrapolation of the map at low speeds. In reality, that discontinuity would not be present as it is a simulation artifact. The discontinuity introduces an apparent delay of approximately 6 s during shutdown and 8 s during startup. Consequently, the reported settling times are conservative; the underlying power response is expected to be slightly faster.

On the other hand, the heat exchanger responses are comparatively slower, especially during shutdown. On average, the heat exchangers reach an idle state in around 520s, whereas they are much faster during startup; reaching their steady state value around on average 30s, except for the cooler, which takes slightly longer at 160s. Oscillations are observed in the CHX temperature trace; these are believed to be numerical artifacts associated with the transient solver and/or property interpolation. As they do not materially affect the power-tracking metrics reported here, a dedicated numerical sensitivity study is left outside the scope of this work.

According to Mongird et al.,^58^ compressed air energy storage and combustion turbine systems, which are comparable to PTES systems due to their dependence on turbomachinery and thermodynamic cycles, exhibit a cold-start response of around 3–10 min. The PTES system studied here exhibit slightly faster response times to startup and shutdown, demonstrating its viability. Additionally, according to a recent reporting of actual electrothermal energy storage system performance demonstration by MAN Energy Solution,^59^ the demonstrated system, that uses sCO_2_ cycles, the ramp up and ramp down times are in the order of 60–90 s. While this is not exactly a precise validation, it shows that the PTES system in this study, demonstrates dynamics comparable to representative systems.

## 5 Discussion

The plantwide control framework developed in this study provides a practical basis for operating PTES systems that are coupled both to renewable electricity systems and to industrial heat users. The power-tracking results show that the system can respond sufficiently quickly for applications such as emergency support, load following, and other short-time scale balancing services. However, the startup results also indicate that a PTES system of this type is not suited to the fastest ancillary services, such as frequency regulation from a cold or idle state. In this respect, PTES is better viewed as a flexible long-duration energy storage technology for capacity support and coordinated heat-and-power management rather than as a substitute for ultrafast-response storage technologies.

From the perspective of industrial heat integration, the dynamic switching results are particularly important. Previous scheduling studies for multitemperature PTES operation assumed idealized transitions between operating modes. In practice, however, such transitions are not instantaneous and must be accommodated explicitly in both control design and operational planning. The present study shows that switching between the high-temperature and medium-temperature operating cycles can be achieved with pressure settling in approximately 48 s and thermal stabilization in approximately 73 s. These values indicate that multitemperature PTES operation is feasible on intrahour time scales and that the system can support industrial heat delivery across multiple temperature levels without requiring excessively slow transitions. More broadly, these transient results provide physically meaningful ramp-rate limits that can be incorporated into future scheduling and grid support studies. In this way, the dynamic modeling and analysis presented in this work informs not only control design, but also system dispatch, storage sizing, and heat-exchanger design under variable operating conditions.

The observed discontinuities during startup and shutdown arise mainly from how the low-speed region of the compressor model is treated. At higher shaft speeds, compressor behavior is represented using the a map (Figure 11), whereas below the validated map range, the model switches to a linear extrapolation to ensure numerically stable spool-up and spool-down behavior. This switching introduces small discontinuities in the power and temperature profiles, particularly around the transition times, *t* = 160 and *t* = 1220. Although these artifacts do not represent physical behavior, the adopted simplified treatment was considered an acceptable compromise, as it allowed the control-relevant dynamics to be captured while maintaining computational efficiency.

Additional oscillations visible in some temperature profiles, particularly during cycle switching, are attributed to numerical effects associated with the stiffness and nonlinearity of the full dynamic model. The final model contains 178 dynamic states and algebraic couplings arising from the closed thermodynamic loop, which makes the simulations numerically demanding. These oscillations were not found to alter the final steady states or the principal control conclusions, but they do affect transient smoothness and computational effort. In repeat simulations, with same conditions, these oscillations are not consistent. Accordingly, they should be interpreted as numerical artifacts rather than as indications of physically unstable operation.

Although the study is based on a specific recuperated sCO_2_ Brayton-cycle PTES configuration with liquid sensible storage, the main control concepts are expected to transfer to other sensible-heat thermodynamic storage systems in which storage-medium flow rates can be actively manipulated. In particular, anti-surge protection, power control, startup/shutdown sequencing, and working-fluid inventory control are likely to remain relevant across a broader class of closed-cycle PTES layouts. By contrast, the direct applicability of the present results to solid-storage or latent-storage systems is limited, since those configurations generally provide fewer degrees of freedom in storage-side flow and temperature control.

## 6 Conclusion

In this work, a dynamic modeling and control framework was developed for a recuperated supercritical CO_2_ Brayton-cycle pumped thermal energy storage system intended for coupled grid support and multitemperature industrial heat delivery. The study focused on the control layer required to operate such a system safely and flexibly under transient conditions, building on prior work on cycle design, storage selection, and operational scheduling. Four key control functions as part of a plantwide control strategy were formulated and assessed: anti-surge protection, power set point tracking, startup and shutdown sequencing, and working-fluid inventory control for cycle switching.

The results show that the proposed control strategy can regulate the PTES system effectively across a range of operating scenarios. Power tracking was achieved with settling times of 15–45 s, depending on step magnitude. Inventory control enabled switching between high-temperature and medium-temperature operating cycles, with pressure stabilization in approximately 48 s and thermal stabilization in approximately 73 s. Startup and shutdown required 91 and 165 s, respectively, while anti-surge control maintained compressor operation within safe limits during severe flow disturbances. Together, these results demonstrate that the system can respond sufficiently quickly for coordinated heat-and-power applications, while also quantifying the transient limits that constrain feasible operation.

More broadly, the study shows that PTES can be operated as a flexible coupling technology between variable renewable electricity and industrial thermal demand at multiple temperature levels. The transient results provide control-relevant ramp-rate information that can be incorporated into future scheduling, dispatch, and techno-economic studies, replacing the idealized switching assumptions often used in higher-level optimization. In addition, the modeling framework can support future design studies aimed at improving cycle selection, storage integration, and process matching for specific industrial applications. A particularly valuable next step would be the codesign of PTES systems and the coupled industrial process so that both heat recovery opportunities and operational flexibility can be assessed in an integrated manner.

## Data Availability

The MATLAB/Simulink project for the simulations can be found at https://github.com/AISL-at-Imperial-College-London/PTES. The project was created using MATLAB R2025.
